# State-of-the-Art Review: Advantages and Disadvantages of Femoral Versus Central Cannulation

**DOI:** 10.1177/15569845251333344

**Published:** 2025-04-22

**Authors:** Paul Werner, Martin Winter, Iuliana Coti, Amila Kahrovic, Martin Andreas, Thomas Haberl, Daniel Zimpfer, Marek Ehrlich

**Affiliations:** 1 Department of Cardiac Surgery, Medical University of Vienna, Austria

**Keywords:** cannulation, cardiopulmonary bypass, cardiac surgery, central cannulation femoral cannulation, percutaneous cannulation

## Abstract

The choice of cannulation technique for cardiopulmonary bypass remains a critical decision in cardiac surgery with direct consequences for intraoperative management and patient outcomes. Central and femoral cannulation represent the 2 dominant approaches, each associated with unique anatomical considerations, hemodynamic implications, and perioperative risks. The correct selection of a cannulation strategy should limit the risk of embolic events and associated complications such as vascular injury and stroke. The purpose of this review is to provide a detailed comparison of central and femoral cannulation techniques, with an emphasis on clinical scenarios and outcomes, recent innovations, and state-of-the-art technology. By critically analyzing current evidence, we aim to offer insights into the optimal cannulation strategy tailored to specific patients.

## Graphical abstract

**Visual abstract:**
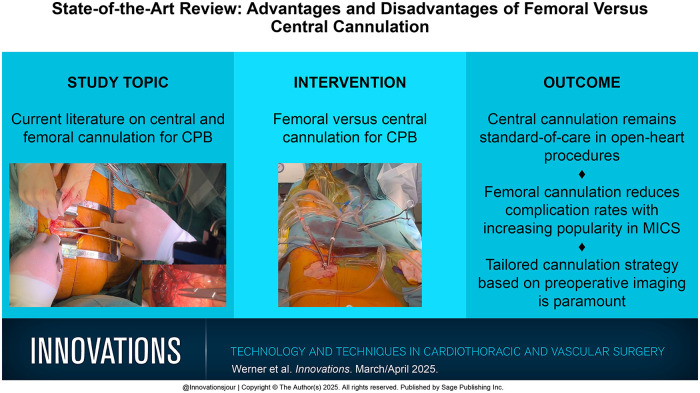

**Central Message**

Central and femoral cannulation present distinct advantages and disadvantages. Central cannulation remains standard in most open-heart procedures, whereas percutaneous femoral approach has become popular in minimally invasive scenarios.

## Introduction

Recent advancements in surgical techniques and perioperative management have spurred renewed interest in optimizing cardiopulmonary bypass (CPB) cannulation techniques. Although central cannulation has traditionally been used in open-heart procedures since the late 1960s, femoral cannulation remains an important option in minimally invasive or urgent cases.^1,2^ Ongoing debates surround the optimal approach for various clinical scenarios.

### Historical Perspective

The evolution of cannulation techniques in CPB during cardiac surgery has undergone significant transformations since the mid-20th century. The introduction of CPB in the 1950s, pioneered by Dr. John Gibbon, marked a crucial milestone in cardiac surgery.^3^ Early cannulation methods were rudimentary and faced challenges due to limited equipment and techniques. The subclavian and the femoral artery were initially used for arterial cannulation in the earliest procedures with CPB.^3^ During the 1960s, direct aortic cannulation was proposed by some groups as a safe and reliable alternative without the need for an additional incision,^4^ which became the standard for most open-heart surgery by the end of the decade.^2^

By the early 2000s, minimally invasive cardiac surgery (MICS) led to reconsideration and further innovations in cannulation strategies. There was a renewal of interest in peripheral cannulation, particularly via the femoral artery, which enabled less invasive approaches over limited accesses with improved patient outcomes. Today, percutaneous cannulation is a widely adopted technique, especially in emergency scenarios, allowing the rapid initiation of CPB.^5^ In addition, technological advancements in imaging and monitoring have improved the precision and safety of modern cannulation techniques.^6^

## Technical Overview of Cannulation Techniques

When comparing central and femoral cannulation techniques, each method presents with distinct advantages and disadvantages, depending on the clinical scenario. Central cannulation is the standard and well-established approach, often associated with predictable outcomes. Although feasible in some minimally invasive approaches, it is the gold standard cannulation associated with a full sternotomy, which itself introduces significant risks such as increased blood loss, postoperative respiratory dysfunction, deep sternal wound infections, and chronic postoperative pain.^7^ Beyond these general risks, the sternotomy itself brings a range of specific sternal complications including mediastinitis, deep sternal abscesses, and sternal osteomyelitis, which are also possible, requiring prolonged antibiotic therapy and potentially additional surgical intervention.^8^ In addition, central cannulation carries a small risk of aortic dissection and the possibility of air or thromboembolism, which can have severe consequences.^9^

On the other hand, femoral cannulation offers a less invasive alternative, providing rapid access, especially in emergencies, and avoiding the need for a sternotomy. This approach is particularly advantageous in patients unsuitable for sternotomy or where no sternotomy is planned (MICS procedures) or if CPB is required before sternotomy.^10^ Peripheral cannulation carries a higher risk of retrograde aortic dissection (0.5%) and can result in a variety of vascular complications. These include distal limb ischemia, femoral artery stenosis, and retroperitoneal hematoma. Postoperative lower limb complications have also been reported, including edema, compartment syndrome, and deep wound infections, which can lead to prolonged recovery times and additional interventions. Access-related complications, such as pseudoaneurysms, groin seromas, and lymphatic fistulas, are additional concerns with this approach. Although peripheral cannulation avoids the significant sternal complications associated with sternotomy, it introduces a different set of risks, particularly in relation to vascular access and postoperative lower extremity complications.^11,12^

### Central Cannulation Technique

**Fig. 1.**
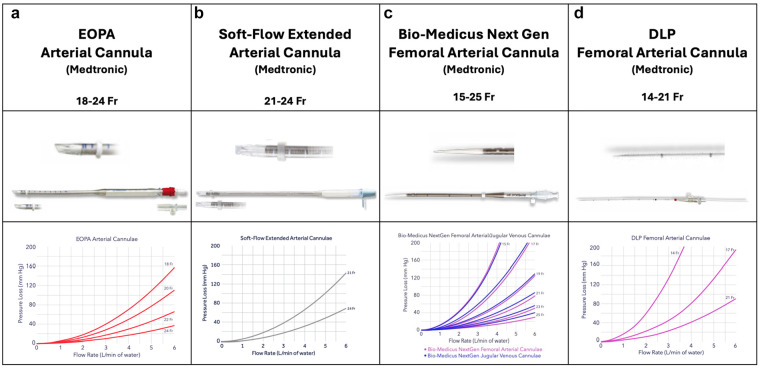
Several cannulas in commercial use for central arterial cannulation: (a) EOPA Arterial Cannula (Medtronic, Dublin, Ireland), (b) Soft-Flow Extended Arterial Cannula (Medtronic), (c) Bio-Medicus Next Generation Arterial Cannula (Medtronic), and (d) DLP Femoral Arterial Cannula (Medtronic). Published with permission from Medtronic.

Central cannulation is typically performed after a median sternotomy. In general, an arterial cannula of sufficient diameter for the calculated pump flow rate is placed into the distal ascending aorta just below the origin of the brachiocephalic trunk. After the placement of 2 purse-string sutures (3-0 polypropylene or 2-0 braided polyester) in the adventitial layer of the aortic wall, the entry site is freed from the adventitia, and a small incision with an 11 or 15 blade is made. The cannula is then carefully introduced into the aortic lumen. For direct insertion, a range of different cannulas can be used, from curved to angled or even straight open tips (Fig. 1). Alternatively, closed-tip cannulas with an introducer can be used for easier insertion (Fig. 1). Some surgeons argue that these carry a lower risk for iatrogenic aortic dissection during cannulation; however, no data on this observation exist in the current literature. Arterial cannulation should be carried out with arterial pressures less than 100 mm Hg to minimize the risk of aortic dissection, and proper positioning should be confirmed, ensuring the tip of the cannula is directed toward the aortic arch.

**Fig. 2.**
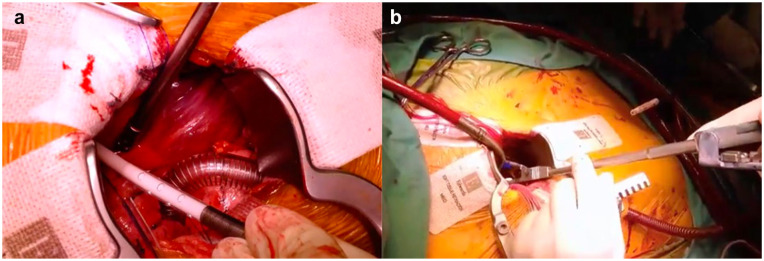
Central cannulation during minimally invasive aortic valve replacement. (a) Arterial cannulation with a Seldinger technique over a right anterior thoracotomy. (b) Placement of the cannula in limited surgical access.

Central cannulation can also be performed applying Seldinger’s technique, which is especially helpful when cannulating the aorta in the aortic arch or in case of minimally invasive procedures with the need for remote aortic cannulation (Fig. 2, Supplemental Video). Needle puncture of the aorta within the purse-string sutures and wire introduction are followed by confirmation of the wire position in the descending aorta. The cannula with introducer is then inserted after serial dilation or directly with a stab incision onto the wire. Confirmation of wire placement in the descending aorta is of utmost importance, as wire displacement in the supra-aortic branches might lead to cannula mispositioning within the carotid or subclavian arteries, with possibly fatal consequences. Hight arterial line pressures after initiation of CPB with low systemic blood pressure should always raise the suspicion of iatrogenic aortic dissection of cannula misplacement within the supra-aortic vessels or positioning of the outflow adjacent to the aortic wall.

### Femoral Cannulation Technique

Femoral cannulation may be achieved via cut down of the femoral vessels or percutaneously. In open femoral cannulation, the procedure begins with a small vertical or oblique incision above the groin crease to surgically expose the common femoral artery. Once the femoral artery is identified and ensnared with vascular loops, two 5.0 polypropylene purse-string sutures are placed longitudinally and secured with tourniquets. A rectangular and longitudinal placement of the purse-string sutures is important to avoid vessel stenosis after decannulation and tying of the sutures. Puncture is performed within the purse-string sutures, and correct wire placement in the descending aorta is confirmed using transesophageal echocardiography (TEE) visualization. The arterial cannula is then inserted over a wire in Seldinger’s technique after subsequent dilatation with increasingly sized dilators or with the aid of a small stab incision onto the wire. After cessation of CPB, the cannulas are removed, and the purse-string sutures are knotted to close the vessels. In small-calibrated vessels, the femoral artery might be ensnared proximal and distal and then incised transversely. The arterial cannula is then introduced gently for a few centimeters into the common iliac artery and fixated with the vessel loop. For distal limb perfusion, a 6 French arterial line is inserted into the distal common femoral artery and also fixated with the snare. Following cessation of CPB, decannulation is performed, and the vessels are reconstructed with single interrupted stitches (6-0 polypropylene sutures).

**Fig. 3.**
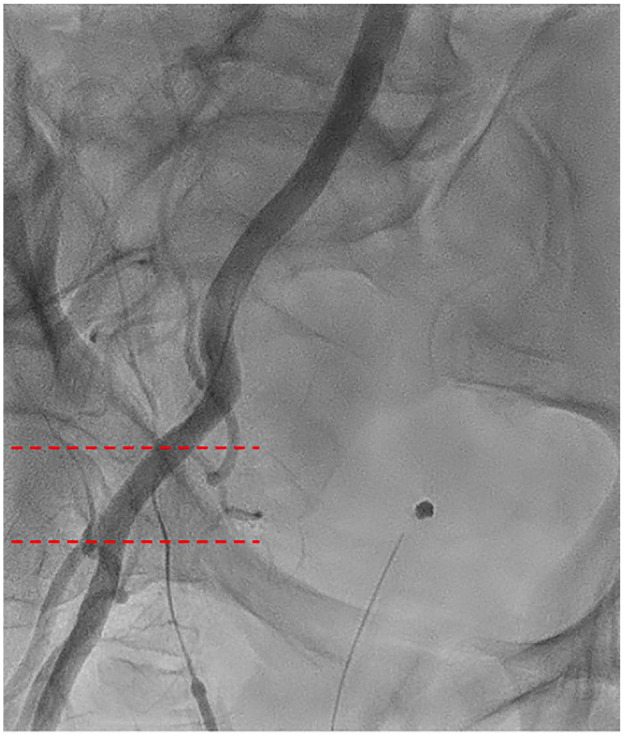
Fluoroscopic imaging of the ideal common femoral artery puncture site between the dotted lines. Vessel entry should always be on the anterior surface in a plaque-free region between the femoral artery bifurcation caudally and the inferior epigastric artery cranially in the middle of the femoral head.

**Fig. 4.**
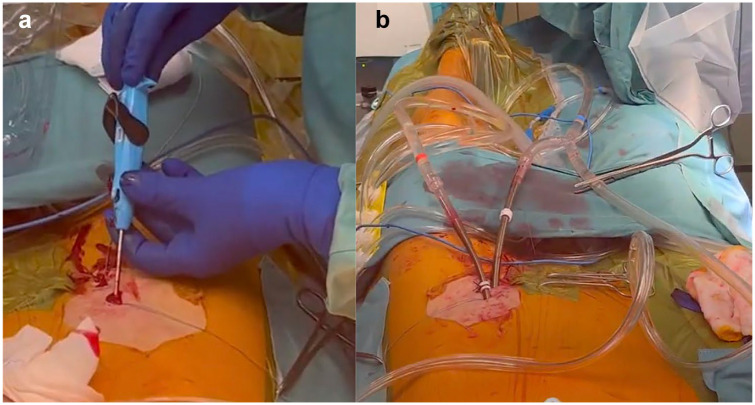
Percutaneous femoral artery cannulation using endovascular closure devices. (a) Endovascular deployment of the ProStyle device (Abbott, Chicago, IL, USA). (b) Final arterial and venous cannula positioning.

Percutaneous femoral cannulation involves accessing the femoral artery and vein without surgical dissection, utilizing the Seldinger technique (Supplemental Video). After needle puncture of the vessels, which should always be performed under sonographic (or fluoroscopic) control, guidewires are inserted under TEE guidance into the descending aorta (Fig. 3). A suture-based endovascular closure device should then be placed in the artery; commonly, 2 ProGlide or ProStyle Systems (Abbott, Chicago, IL, USA) are used with 10 and 2 o’clock orientation (Fig. 4a). The vessel is then dilated, and the arterial cannula is placed and secured (Fig. 4b). This device places a suture through the anterior wall of the vessel, which is tightened after removal of the cannula. Once CPB is weaned and the cannulas are removed, vessel closure is performed using the pre-placed suture-mediated closure devices, followed by manual compression if necessary. To prevent leg ischemia due to femoral artery occlusion by the arterial cannula, a distal leg perfusion cannula can be used to maintain adequate limb perfusion.^13^ Importantly, the risk of wound infection or seroma due to lymphatic fistula is almost completely avoided by percutaneous cannulation.

### Cannulation Strategies for MICS

In MICS, femoral cannulation is commonly preferred as it maintains a clear operative field, facilitating smaller incisions such as minithoracotomies with limited fields of vision. The femoral artery is easily accessible and generally of sufficient caliber to accommodate a large cannula, ensuring optimal flow dynamics. Careful patient selection aided by preoperative computed tomographic angiography is mandatory to identify individuals with peripheral vascular disease or atheromatous aortas and helps mitigate the risks of retrograde flow and embolic events, enabling safe and effective CPB.^14^

Conversely, central aortic cannulation, which provides antegrade flow more closely resembling physiological perfusion, is typically reserved for cases in which femoral access is contraindicated or impractical. This approach offers direct access to the aorta, making it advantageous for patients with significant peripheral vascular disease, where retrograde flow may elevate the risk of embolization, especially for patients with aortic valve disease and higher calcium load in the peripheral vessels and descending aorta. However, in the context of MICS through a thoracotomy, aortic cannulation is less frequently used due to space limitations for instrumentation and the challenges of positioning the cannula within the operative field.^14,15^ Nevertheless, central cannulation, similar to its use in open sternotomy procedures, is often favored when a hemisternotomy is performed, particularly in minimally invasive aortic valve replacement.^16^ At our institution, central aortic cannulation has also become a standard for aortic valve replacement through an anterior right thoracotomy, as a nonnegligible portion of these patients present with peripheral vascular disease (Fig. 2).

### Different Scenarios in Acute Type A Aortic Dissection

In the management of acute type A aortic dissection (ATAAD), the choice of cannulation is determined by various patient-specific factors, including hemodynamic stability, the extent of the dissection, and the planned scope of surgical repair, alongside the risks and benefits associated with each method. Central cannulation, typically via the ascending aorta, offers the significant advantage of antegrade perfusion, which better supports physiological blood flow. However, there is a risk of inadvertently cannulating the false lumen of the dissected aorta, potentially leading to progression of the dissection or even aortic rupture.^17^

On the other hand, femoral cannulation is frequently chosen for its technical simplicity and speed, particularly in patients who are hemodynamically unstable. The femoral artery provides quick and accessible entry for the initiation of CPB, which is critical in emergency situations.

Although not the primary focus of this review, axillary cannulation has seen increasing use in recent years. The axillary artery is often unaffected by dissection, making it a safer option in many cases. Moreover, axillary cannulation is particularly beneficial for maintaining antegrade cerebral perfusion. However, axillary cannulation is more time-consuming, which can be a significant drawback in unstable patients who require rapid initiation of CPB.^18^

### Different Scenarios in Redo Procedures

In reoperative cardiac surgery, one of the most critical aspects of the procedure is safely navigating the sternal reentry and managing the adhesions and scar tissue from previous surgeries. The risk of injuring vital structures, such as the aorta, right ventricle or atrium, right ventricular–pulmonary artery conduits, or bypass grafts that may adhere to the posterior surface of the sternum, is significantly higher in reoperations. These challenges make the choice of timing for CPB crucial for optimizing procedural success. Femoral cannulation, which can be used to initiate CPB early before redo sternotomy, is often used in cases with unfavorable anatomy of cardiac structures directly adhering to the posterior border of the sternum. Early CPB initiation immediately decompresses the heart and great vessels, limiting the risk of injury before reentry. In the event of injury, a cardiotomy sucker can be used instantly to manage blood loss and reinfuse the bleeding volume.^19^

However, in low-risk cases, early CPB, which implies the need for full heparinization, may not be necessary and might increase the operative risk based on coagulopathy due to prolonged CPB duration. In situations with stable hemodynamics and mediastinal structures nonadherent to the sternum, central and therefore late CPB cannulation can be employed.^10,19^ When confronted with “intermediate risk” anatomies, another option is to perform femoral puncture and insertion of the guidewires under echocardiographic control and then proceed with the sternotomy. In the case of reentry injury, immediate femoral cannulation can then be rapidly performed.

### Current Guidelines

The European Association for Cardio-Thoracic Surgery 2019 guidelines on cannulation for CPB emphasize the importance of site selection based on clinical context and patient risk factors. The distal ascending aorta remains the most common arterial cannulation site. In emergencies requiring rapid CPB, such as cardiac arrest, aortic dissection, or severe bleeding, femoral artery cannulation is the preferred choice. It is also recommended in reoperative and MICS. The axillary–subclavian artery is gaining favor, particularly in aortic dissection, due to its capacity to allow antegrade flow and reduced atherosclerotic risk. The guidelines offer a Class IIb recommendation for the use of epiaortic ultrasound to identify plaques prior to aortic cannulation. This technique is particularly advised for patients over the age of 50 to 60 years, especially those with a history of stroke, transient ischemic attacks, peripheral artery disease, or calcifications noted in prior imaging studies.^20^

## Comparative Analysis of Cannulation Techniques

### Flow Direction and Flow Reversal During CPB

While central aortic cannulation preserves the physiological state of antegrade systemic arterial perfusion, femoral cannulation creates a retrograde flow in the descending aorta. The effects of arterial flow reversal during extracorporeal circulation on a cellular level have not been well described. It has been shown that endothelial cells are able to detect and respond to changes in flow direction within the vessel, measurable via nitric oxide synthesis and inflammatory pathway activation.^21^ Nitric oxide synthesis is at maximum when the cells are aligned in the flow direction and at lowest when their cytoskeleton is perpendicular to the blood flow. Recent results from small animal models also observed increased cytokine levels in retrogradely perfused vessels.^22^ Clinical data from the Framingham study showed that in larger arteries (brachial artery), flow reversal was accompanied by impaired nitric oxide production and vasodilator function.^23^ Currently, the clinical effect of retrograde systemic perfusion regarding inflammation is unknown.

On a macroscopic level, retrograde perfusion in the descending aorta and the aortic arch might lead to retrograde arterial embolization of atherosclerotic plaques. Due to the sudden change in flow direction, the differently oriented shear stress on an aortic plaque or an intraluminal thrombus might carry it away. This can result in catastrophic complications such as stroke. The group from New York University reported early results with mitral valve (MV) MICS procedures, where they identified peripheral vascular disease as a predictor for neurologic events.^24^ In a subsequent analysis, they identified retrograde arterial perfusion as a significant predictor for neurologic events (odds ratio [OR] = 3.8, *P* = 0.01).^25^ These results were confirmed by Murzi and colleagues from Massa, Italy, who showed that retrograde perfusion in MV surgery was associated with a significantly increased incidence of strokes in comparison with antegrade systemic perfusion (5% vs 1%, *P* = 0.002).^26^ Bedeir et al. also observed an increased risk of stroke in their retrospective study in the MICS MV repair group in comparison with the sternotomy group.^27^ A meta-analysis by Cheng et al. investigating MICS MV surgery versus open (full sternotomy) MV surgery yielded similar results, with an increased risk of stroke in the MICS cohort.^28^

In ATAAD, the natural blood flow is already impaired due to pathological morphological changes of the aorta, and surgery for ATAAD aims to restore physiological blood flow to avoid ischemia. Femoral artery cannulation for CPB initiation is a possible strategy in ATAAD surgery; however, the exact flow distribution and subsequent malperfusion due to false lumen perfusion is not predictable. Although femoral (peripheral) cannulation gives a relatively safe option for arterial cannulation in terms of access reliability and bleeding risk, the flow reversal can promote false lumen perfusion with true lumen collapse and subsequent malperfusion leading to neurologic sequelae. Results from a large meta-analysis support this; Benedetto et al. included 14 studies with a total of 4,476 patients, investigating the impact of arterial cannulation site on permanent neurologic deficit and postoperative morbidity after thoracic aortic surgery.^29^ They showed that central cannulation was protective for in-hospital mortality in contrast to femoral cannulation (risk ratio [RR] = 0.59, 95% confidence interval [CI]: 0.48 to 0.7, *P* < 0.001) and for permanent neurologic deficits (RR = 0.71, 95% CI: 0.55 to 0.90, *P* = 0.005). When comparing femoral with axillary cannulation (which is considered more central), data also hint toward improved neurologic outcomes in ATAAD with axillary cannulation; however, the current literature suffers from a lack of randomized trials, comparison of historical cohorts with time bias, and small cohort sizes. A recent meta-analysis from Ren and colleagues investigated 9 studies and observed a significantly reduced risk for short-term mortality (OR = 0.25, 95% CI: 0.15 to 0.42, *P* < 0.001) and incidence of neurologic adverse events (OR = 0.46, 95% CI: 0.29 to 0.72, *P* < 0.01) in favor of the axillary artery group.^30^ Not all published literature supports the superiority of central (and axillary) cannulation in terms of neurologic events; some studies have found no superiority of any cannulation site,^31^ and a most recent meta-analysis from the Pittsburgh group in more than 14,000 patients showed a trend toward improved outcomes with central cannulation, which did not reach statistical significance.^32^

### Vascular and Access Site–Related Complications

Arterial cannulation for CPB involves manipulation of large vessels, which bears the risk of vascular complications such as iatrogenic (retrograde) dissection, bleeding, pseudoaneurysms, and formation of hematoma. The risk for vascular complications in central aortic cannulation is generally considered low but serious when observed. Rates for iatrogenic aortic dissection in cardiac procedures range from only 0.06% to 0.23%; however, its occurrence is associated with considerable mortality from 15% up to 50% in reported series.^9,33,34^ When confronted with iatrogenic aortic dissection, immediate recognition of the problem is paramount. After cessation of CPB over the affected arterial site, a new arterial cannula should be introduced at a different, nonaffected location (femoral, axillary, arch). Immediate aortic repair should then commence, followed by completion of the originally planned procedure. As the exact location of the entry tear is known, a localized repair strategy can be pursued, mostly consisting of an aggressive hemiarch repair with excision of the affected ascending aortic segment or at least an open distal anastomosis.

Femoral cannulation might also lead to (retrograde) iatrogenic aortic dissection, which is a significantly higher risk than in central aortic cannulation, ranging from about 0.47% in comparison with 0.06% in central cannulation.^35^ Predisposing factors are small groin vessels, atherosclerotic disease of the iliofemoral arteries, high arterial line pressures to inadequately chosen cannula size, and incorrect surgical technique. When being confronted with retrograde iatrogenic dissection, several approaches have been described. In the case of early recognition, immediate cessation of CPB and retrograde perfusion with reinfusion of the volume and abortion of the cardiac procedure might be a possible method of treatment.^35^ However, if cessation of CPB is not possible and the ascending aorta is affected, true lumen cannulation of the aorta (Seldinger technique with echocardiographic guidance) and restoration of CPB with subsequent aortic repair should be performed.

Bleeding is a relevant complication, often associated with an increase in postoperative morbidity and mortality. In central cannulation, bleeding might be observed but generally possesses no great challenge as it is usually manageable with suture repair. However, excessive bleeding from the cannulation site before initiation of CPB can be problematic and should always raise the suspicion of iatrogenic aortic dissection. In this case, arterial cannulation should be performed at a different location with subsequent initiation of CPB bypass and aortic repair.

Bleeding events and other vascular complications such as pseudoaneurysms, hematoma, retroperitoneal hemorrhage, arteriovenous fistulas, and arterial stenosis are limited to the femoral artery and are almost never observed in central cannulation due to differences in anatomy. Femoral cannulation has traditionally been performed open via a small groin incision, which limited the risk of arteriovenous fistulas, pseudoaneurysms, and postoperative arterial stenosis. The advent of percutaneous cannulation techniques, however, also increased the risk for these events. In patients after transfemoral transcatheter valve replacement, which also involves percutaneous arterial insertion of large sheaths and endovascular closure, rates for femoral artery stenosis were 9.8%, 2.3% for pseudoaneurysms, 4% for vessel dissection, 0.8% for arteriovenous fistulas, and 9.2% for hematoma.^36^ In patients who underwent femoral cannulation for venoarterial extracorporeal membrane oxygenation (VA-ECMO), the incidence of vascular complications was reported up to 10% in some cohorts,^37^ which included femoral artery perforation, femoral artery dissection, limb ischemia, and hematoma. Recent studies compared percutaneous with surgical techniques for peripheral CPB and showed that the percutaneous technique was associated with lower rates of complications, which was mainly carried by higher incidences of wound infections and seromas but not vascular complications.^38,39^ Saeed et al. reported rates of 0.2% for iatrogenic ATAAD in the surgical cutdown group and 1.1% in the percutaneous group and 0.5% for hematomas in the surgical cutdown group and 0% in the percutaneous group with no other vascular complication observed.^38^ A different study comparing both methods of femoral cannulation for CPB reported complication rates of 1.1% hematoma, 1.1% femoral vessel stenosis in the surgical cutdown group and 0.3% hematoma, 0.8% femoral stenosis, 0.6% arteriovenous fistula or pseudoaneurysm, and 0.6% vascular injury.^39^ Lymphatic fistulas and wound infections were almost exclusively observed in the surgical cutdown groups, ranging from 2.5% for wound infections up to 6.5% for lymphatic fistulas.^11,38,39^ The results for vascular complications in peripheral CPB compare favorably to the literature on transcatheter aortic valve replacement, which might be associated with the limited comorbidity of the patient collective and caused by underreporting due to a lack of standardized postoperative diagnostics.

### Reoperative Cardiac Surgery

**Fig. 5.**
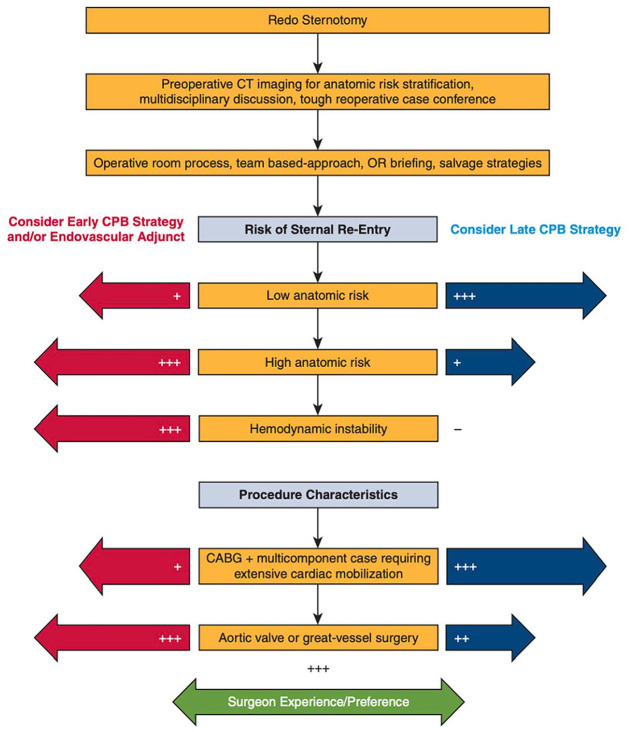
Algorithm for CPB strategies (central vs peripheral or early vs late) in reoperative cardiac surgery as proposed by the Cleveland Clinic. Reprinted from *J Thorac Cardiovasc Surg*, Volume 164, Kindzelski et al., “Modern practice and outcomes of reoperative cardiac surgery,” pages 1755–1766, Copyright 2022, with permission from Elsevier.^10^ CABG, coronary artery bypass grafting; CPB, cardiopulmonary bypass; CT, computed tomography; OR, operating room.

Peripheral cannulation in reoperative surgery is often used to avoid catastrophic injury during sternal reentry, whereas central CPB cannulation is often performed in low-risk cases. In larger cohorts of reoperative cardiac surgery patients, peripheral CPB initiation before sternotomy was used in 26% of patients (*n* = 158), and damage to cardiac structures was observed in 4 patients.^40^ In all cases, injury and blood loss could be managed well due to already established CPB. They concluded that a liberal approach toward early CPB before sternotomy should be pursued. A retrospective study from a group from Istanbul in 258 redo patients compared outcomes between peripheral (before sternotomy) and central (after sternotomy) cannulation groups. A significantly higher incidence of cardiac injury (8.3% vs 1.8%, *P* = 0.038) and red blood cell requirement (*P* = 0.004) was observed in the central cannulation group, yet operative mortality and 1-year survival did not differ significantly between groups.^19^ But not all evidence is in favor of peripheral cannulation before sternotomy. A recent study from a different group from Istanbul performed a 1:1 propensity score matching in a 257 patient cohort, and they found no differences in major cardiac injury or early mortality, with a higher incidence of renal failure in the peripheral cannulation group.^41^ A larger reoperative cohort from the Cleveland Clinic group showed no differences in operative mortality between early (peripheral) and late (central) cannulation in the low or high anatomic risk cohorts.^10^ They advocate for an individualized approach including patient factors and surgeon preferences in absence of superiority of any cannulation strategy and proposed an algorithm as a decision aid (Fig. 5). Brown et al. retrospectively observed long-term outcomes in patients after redo cardiac surgery, of whom 5.5% (*n* = 91) underwent peripheral cannulation.^42^ They observed significantly higher rates of operative mortality in the peripheral cannulation group (18.7% vs 7.5%, *P* < 0.001) and following exclusion of all operative mortalities a significantly reduced long-term mortality (log rank, *P* = 0.047) in the peripheral group, as well as numerically higher rates of blood transfusions (55.6% vs 64.8%, *P* = 0.086). Although the peripheral cannulation group was heterogenous with more urgent/emergent cases and high-risk procedures, peripheral cannulation remained an independent predictor of postoperative mortality in the multivariable Cox proportional hazards regression model (hazard ratio = 1.53, 95% CI: 1.01 to 2.30, *P* = 0.44).

## Emerging Technologies and Techniques

Various studies have reinforced the benefits of percutaneous cannulation. Wang et al. compared outcomes between percutaneous and surgical cannulation in patients undergoing VA-ECMO for cardiac shock, demonstrating that percutaneous cannulation was associated with significantly lower rates of in-hospital mortality (OR = 0.76, 95% CI: 0.70 to 0.84, *P* < 0.01), cannulation site bleeding (OR = 0.70, 95% CI: 0.60 to 0.80, *P* < 0.01), and systemic infection (OR = 0.63, 95% CI: 0.54 to 0.74, *P* < 0.01).^43^ A large meta-analysis similarly found that surgical cannulation poses a significantly higher risk of complications, such as any groin access site complications (OR = 3.09, 95% CI: 1.87 to 5.10, *P* < 0.01), wound complications (OR = 10.10, 95% CI: 3.31 to 30.85, *P* < 0.01), and lymphatic complications (OR = 9.37, 95% CI: 2.15 to 40.81, *P* < 0.01), alongside a longer procedural duration (*P* < 0.01). However, perioperative mortality, vascular complications, femoral stenosis, and stroke were comparable between the 2 approaches.^44^

Percutaneous femoral cannulation necessitates the use of arterial closure devices, with several commercially available options, including suture-based devices such as the Perclose ProStyle and its predecessor the Perclose ProGlide, collagen-based systems such as the MANTA™ (Teleflex Inc., Wayne, PA, USA), and patch-based technologies such as PerQseal® (Vivasure, Galway, Ireland). A recent propensity-matched analysis comparing the MANTA and Perclose ProGlide vascular closure devices in patients undergoing transfemoral transcatheter aortic valve replacement found similar deployment success rates (*P* = 1.000) and mortality rates (*P* = 0.316). Notably, no patients experienced major vascular complications with either device.^45^ Likewise, a comprehensive meta-analysis demonstrated that both collagen-based and suture-based devices offered comparable safety profiles.^46^ Barbash et al. reported that Prostar XL (Abbott) had a significantly higher rate of major vascular complications (16.7%) compared with Perclose ProGlide (3.2%, *P* < 0.001), alongside longer hospital stays (*P* = 0.007), although in-hospital mortality was similar for both devices.^47^ In a large multicenter study, the MANTA device achieved technical success in 97.7% of patients; major vascular complications occurred in 4.2% of patients, including access site bleeding (1.1%), covered stent placement (1.5%), and surgical repair or balloon inflation (0.8%).^48^ We suggest that a ProGlide first strategy is safe, as insufficient closure with 2 ProGlide devices can still be addressed with a MANTA device if the wire is left in place, whereas a failed MANTA closure cannot be address by ProGlide thereafter.

Percutaneous femoral artery cannulation also plays a key role in establishing CPB in MICS, offering reduced surgical trauma and faster recovery times.^49^ However, prolonged CPB procedures may lead to limb ischemia due to reduced blood flow to the lower extremities.^50^ To address this issue, the Bi-Flow™ cannula (LivaNova, London, UK) has been developed as an innovative solution, by providing a dual orifice system without the need for an additional cannula.^51,52^ A randomized controlled trial confirmed that bidirectional cannulation maintains stable distal perfusion throughout the procedure, highlighting its safety and efficacy.^53^

During minimally invasive surgery in patients with peripheral vascular disease, we currently evaluate the usage of a long femoral arterial cannula, inserted until the aortic arch, allowing an almost natural blood flow with groin cannulation. We believe that this reduces the risk of femoral cannulation as it obviates the patients from retrograde perfusion and associated complications. Although the implantation might be more technical demanding than a short femoral cannula positioning, this simple technique might be the next technical advancement in femoral cannulation to reduce cerebrovascular incidents.

For central cannulation, evolution in cannula design, such as the EOPA 3DTM Arterial Cannulae (Medtronic, Dublin, Ireland; Fig. 1) or the Soft-Flow cannula (Medtronic; Fig. 1), offer improvements in blood flow dynamics. The tip of the EOPA 3DTM cannula is designed with 3 integrated flutes, which allow for blood to be dispersed through multiple outlets. This diffusion of blood flow reduces exit velocity, promoting more efficient perfusion. Other studies investigating differences in fluid dynamics based on the cannula outflow showed that subtle changes in design lead to significant differences in hemodynamics. An example for this would be the experimental opti²CAN, which aims to reduce wall shear stresses and thereby possibly lower the risk of plaque dislodgement and subsequent stroke.^54^

## Conclusions

The advent of MICS within the past 2 decades has led to a renewal of interest in femoral cannulation techniques. With this new focus, there was also a rediscovery of old clinical problems associated with retrograde perfusion, such as embolism or dissection. However, a standardized application of modern preoperative imaging nowadays allows for improved patient selection and a personalized approach for arterial cannulation, selecting optimal patients who are good candidates for peripheral CPB to optimize outcomes. The direct transfer of percutaneous techniques from interventional cardiology to peripheral CPB cannulation led to a significant decrease in access site–related complications. Further, improved surgical technique for central cannulation made it also a feasible or even the preferred option for certain minimally invasive procedures such as aortic valve procedures. In acute aortic dissection, central or supra-aortic vessel cannulation might be the preferred option compared with femoral cannulation with regard to postoperative survival and neurologic outcome, yet data on this topic are still not unequivocal. For other scenarios such as reoperative cardiac surgery, individualized concepts based on the patient’s anatomical risk should be applied, as there is no consensus in the literature favoring central or femoral cannulation. There is a gap of evidence due to missing randomized trials in emergent, reoperative, and MICS, concerning differences in outcomes based on arterial cannulation techniques, which should be the focus of future trials.
